# Wheat WW Domain-Containing Protein TaCFL1 Negatively Regulates Cuticular Wax Biosynthesis

**DOI:** 10.3390/ijms252313187

**Published:** 2024-12-08

**Authors:** Wanzhen Chen, Lang Liu, Xiaoyu Wang, Haoyu Li, Jiao Liu, Pengfei Zhi, Cheng Chang

**Affiliations:** College of Life Sciences, Qingdao University, Qingdao 266071, China

**Keywords:** wheat, WW domain-containing protein, class IV homeodomain transcription factor, 3-Ketoacyl-CoA synthase, wax biosynthesis

## Abstract

Waxy cuticle covers plant aerial organs and protects plants against environmental challenges. Although improved cuticle-associated traits are aimed at the wheat breeding programs, the mechanism governing wheat cuticular wax biosynthesis remains to be elucidated. Herein, wheat WW domain-containing protein TaCFL1 is characterized as a negative regulator of wax biosynthesis. The knockdown of *TaCFL1* expression results in a 15% increase in wax accumulation and decreased leaf cuticle permeability in bread wheat. Furthermore, wheat class IV homeodomain transcription factors TaHDG1.1 and TaHDG1.2 are identified as partially redundant activators of wax biosynthesis. The silencing of *TaHDG1.1* or *TaHDG1.2* expression leads to an 11% reduction in epidermal wax accumulation and an increase in leaf cuticle permeability wax, while the co-silencing of *TaHDG1.1* and *TaHDG1.2* results in a 31% reduction in epidermal wax accumulation and a further increase in wax in the leaf cuticle permeability. Moreover, wheat 3-Ketoacyl-CoA synthase TaKCS10 is isolated as an essential component of the wax biosynthetic machinery. The silencing of *TaKCS10* expression results in a 22% reduction in wax accumulation and increased leaf cuticle permeability. In addition, we demonstrated that the *TaKCS10* expression is activated by TaHDG1.1 and TaHDG1.2, and that TaCFL1 attenuates the TaHDG1-mediated transcriptional activation of *TaKCS10*. This evidence supports that the WW domain-containing protein TaCFL1 negatively regulates wax biosynthesis via attenuating the transcriptional activation of the *TaKCS10* gene mediated by HD-ZIP IV transcription factor TaHDG1.

## 1. Introduction

As an adaptive innovation during the plant transition from water to land, the cuticle seals plant aerial organs like leaves, stems, and flowers at the stage of primary growth [1,2,3,4,5]. The hydrophobic cuticle restricts nonstomatal water loss and limits gas exchanges with surroundings, contributing to plant adaptation to various abiotic stresses like drought, salinity, UV-B radiation, and extreme temperatures [6,7,8,9]. As the first contact site between plant aerial organs and invading pathogens and pests (P&Ps), the cuticle plays multifaceted roles in the regulation of plant interaction with adapted P&Ps [10]. For instance, silencing the wheat wax biosynthesis gene *Enoyl-CoA Reductase* resulted in the reduced germination of the adapted wheat powdery mildew, *Blumeria graminis forma specialis tritici* [11]. In addition, the cuticle affects multiple developmental events [12]. Indeed, leaf deformations and fusion were observed on the *Arabidopsis thaliana* cuticle biosynthetic mutant *bodyguard* (*bdg*) [13]. Beneficial cuticle-associated traits positively contributing to crop quality and quantity have been widely employed in crop breeding [14,15]. Increased leaf wax alkane concentration has been selected in the breeding efforts of bread wheat (*Triticum aestivum* L.) [16].

Although chemical compositions and structures of lipids in the cuticle vary among plant species and organs, the lipophilic cuticle is mainly composed of cutin and wax [17,18]. Cutin is organic solvent-insoluble and mainly composed of cross-linked polyesters of long-chain (C16 or C18) oxygenated fatty acids [17,18]. In contrast, wax mixtures are organic solvent-extractable and mainly consist of very-long-chain (VLC, above C20) fatty acids and their derivatives such as alcohols, alkanes, aldehydes, alkenes, ketones, and esters [17,18]. Cutin and wax are synthesized in the endoplasmic reticulum (ER) of plant epidermal cells by modifying C16 and C18 fatty acyl-CoAs [19,20,21,22,23,24,25,26,27]. For the wax biosynthesis, fatty acid elongase (FAE) complexes composed of Ketoacyl-CoA synthases (KCS), Ketoacyl-CoA reductases (KCR), hydroxyacyl-CoA dehydratases (HCD), and enoyl-CoA reductases (ECR) catalyze the aliphatic chain elongation of C16 and C18 acyl-CoAs to form VLC acyl-CoAs [28,29,30,31,32,33,34,35]. VLC alkanes, aldehydes, ketones, and secondary alcohols are modified from VLC acyl-CoAs in the alkane-forming pathway [36,37,38,39,40,41,42]. VLC primary alcohols and esters are converted from VLC acyl-CoAs by an alcohol-forming pathway [43,44,45]. These wax constituents are then exported out of the ER, cross the plasma membrane, and finally deposited to the extracellular cuticular regions [46,47,48,49,50,51,52,53,54,55,56,57]. Although the biosynthesis of wax is extensively studied in the dicot model plant *Arabidopsis thaliana*, its mechanism in the crop wheat remains to be elucidated [14,21,22].

The biosynthesis of wax has been demonstrated to be regulated by transcription factors in the model dicot Arabidopsis [21,22]. For instance, Arabidopsis APETALA2/ethylene response factor (AP2/ERF)-type transcription factors SHINE1/WAXINDUCER1 (AtSHN1/WIN1), AtSHN2, and AtSHN3 positively regulate the accumulation of wax, whereas AP2/ERF-type transcription factor DEWAX negatively regulates wax biosynthesis [58,59,60,61,62,63,64]. Arabidopsis myeloblastosis (MYB)-type transcription factors AtMYB16, AtMYB30, AtMYB94, AtMYB96, and AtMYB106 are identified as positive regulators of wax biosynthesis [65,66,67,68,69,70,71]. Class IV homeodomain leucine zipper (HD-ZIP IV) transcription factor AtHDG1 positively regulates Arabidopsis wax biosynthesis by activating wax biosynthesis genes *FIDDLEHEAD* (*AtFDH*) [72]. Arabidopsis WW domain-containing protein AtCFL1 is demonstrated to negatively regulate cuticle development by affecting the function of AtHDG1 [72]. However, whether and how WW domain-containing proteins and HD-ZIP IV transcription factors become involved in the regulation of wheat wax biosynthesis remains unknown.

In this study, wheat WW domain-containing protein TaCFL1 is characterized as a negative regulator of wax biosynthesis. Silencing of *TaCFL1* results in increased wax accumulation and decreased leaf cuticle permeability. Furthermore, wheat class IV homeodomain transcription factors TaHDG1.1 and TaHDG1.2 are identified as partially redundant activators of wax biosynthesis. The silencing of *TaHDG1.1* or *TaHDG1.2* expression results in reduced accumulation of wax and enhanced leaf cuticle permeability, while the co-silencing of *TaHDG1.1* and *TaHDG1.2* leads to a further decrease in the accumulation of wax and a further increase in the leaf cuticle permeability. Moreover, wheat 3-Ketoacyl-CoA synthase TaKCS10 is isolated as an essential component of wax biosynthetic machinery. The silencing of *TaKCS10* expression results in decreased accumulation of wax and increased leaf cuticle permeability. In addition, we demonstrated that *TaKCS10* expression is activated by TaHDG1.1 and TaHDG1.2, and TaCFL1 attenuates the TaHDG1-mediated transcriptional activation of *TaKCS10*. Therefore, this study revealed that the WW domain-containing protein TaCFL1 negatively regulates wax biosynthesis, probably via attenuating the transcriptional activation of the *TaKCS10* gene mediated by the HD-ZIP IV transcription factor TaHDG1.

## 2. Results

### 2.1. Identification of Wheat TaCFL1 Based on Homology with Arabidopsis AtCFL1 Protein

**Figure 1:**
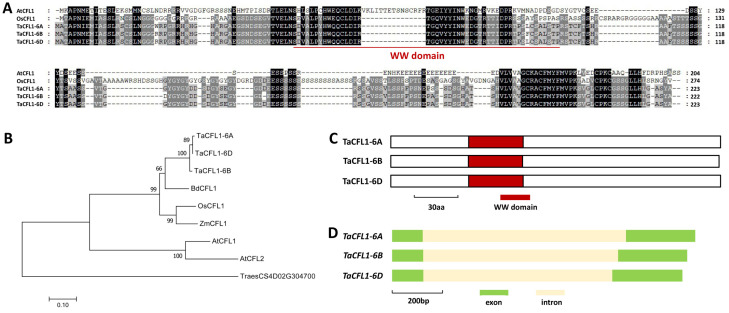
Identification of wheat TaCFL1 based on homology with Arabidopsis AtCFL1. (**A**) Protein sequence alignments of wheat TaCFL1, Arabidopsis AtCFL1, and rice OsCFL1. Conserved residues among five protein sequences are shaded in dark, while residues conserved in at least three of the five proteins are shaded in gray. (**B**) Phylogenetic relationships of the CFL1 homologs from *Arabidopsis*, *Brachypodium*, maize, rice, and wheat. Two-letter genus-species prefixes: At, *Arabidopsis thaliana*; Bd, *Brachypodium distachyon*; Os, *Oryza sativa*; Ta, *Triticum aestivum*; Zm, *Zea mays*. (**C**) Domain structures of wheat TaCFL1 proteins. (**D**) Gene architectures of wheat *TaCFL1* genes.

In this study, we are interested in exploring the function of the WW domain-containing protein TaCFL1, the wheat homolog of Arabidopsis AtCFL1, in the regulation of wheat wax biosynthesis. We first employed the Arabidopsis AtCFL1 (At2g33510) as a query to search for the wheat reference genome. Wheat TaCFL1 was identified as the closest homolog of Arabidopsis AtCFL1. As shown in Figure 1A, *TaCFL1-6A* (*TraesCSU02G080500*), *TaCFL1-6B* (*TraesCSU02G087700*), and *TaCFL1-6D* (*TraesCS6D02G181600*) are highly homologous sequences of the TaCFL1 gene located on wheat chromosomes 6A, 6B, and 6D (Supplemental Figure S1). As shown in Figure 1A, TaCFL1-6A, TaCFL1-6B, and TaCFL1-6D proteins shared above 34% amino acid sequence identities with Arabidopsis AtCFL1 and rice OsCFL1 proteins. Phylogenetic analysis confirmed that wheat TaCFL1 is the closest homolog of Arabidopsis AtCFL1 and AtCFL2 (Figure 1B). One WW domain was identified from TaCFL1-6A, TaCFL1-6B, and TaCFL1-6D proteins (Figure 1C). As shown in Figure 1D, the gene architecture of two exons and one intron is consistent across all three *TaCFL1* genes (6A, 6B, and 6D).

### 2.2. Functional Analysis of Wheat TaCFL1 in the Wax Biosynthesis

**Figure 2:**
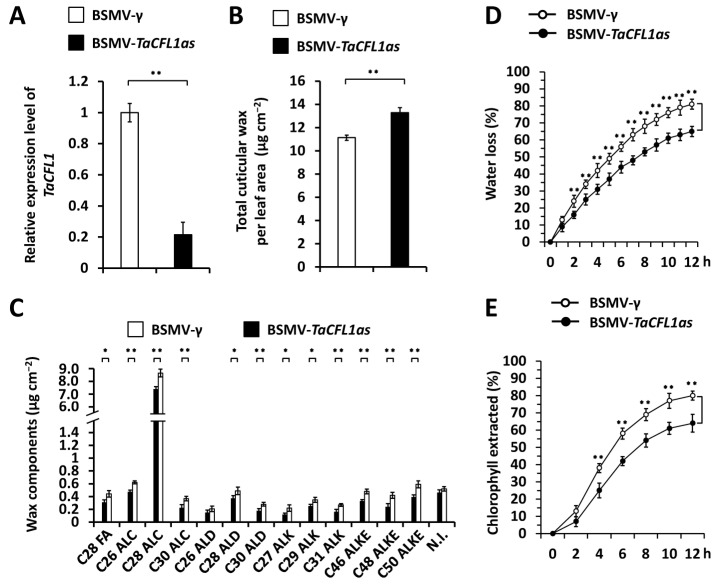
Functional analyses of wheat *TaCFL1* genes in the accumulation of leaf wax. (**A**) qRT-PCR analysis of *TaCFL1* expression levels in the wheat leaves silencing *TaCFL1* (BSMV-*TaCFL1as*). (**B**) Loads of wax on the wheat leave silencing *TaCFL1*. (**C**) Amounts of major wax components in the wheat leaves silencing *TaCFL1*. FA, fatty acid; ALC, alcohol; ALD, aldehyde; ALK, alkane; ALKE, alkyl ester; N.I., not identified compound. (**D**) Water loss rates and (**E**) chlorophyll extraction levels were analyzed in wheat leaves silencing *TaCFL1.* For (**A**–**E**), leaves of wheat plants infected with BSMV-*γ* were employed as the negative control, and three independent biological replicates per treatment were statistically analyzed, and data are presented as the mean ± SE (Student’s *t*-test; * *p* < 0.05, ** *p* < 0.01).

To explore the potential regulation of wheat WW domain-containing protein TaCFL1 on the accumulation of wax, we silenced all endogenous *TaCFL1* genes using the barley stripe mosaic virus-induced gene silencing (BSMV-VIGS) in the plants of wheat cultivar Yannong 999. It was previously demonstrated that BSMV infection has no significant effect on the wax deposition in bread wheat [11]. As revealed by the quantitative reverse transcription–polymerase chain reaction (qRT-PCR) assay, the expression level of the *TaCFL1* gene was significantly reduced in wheat leaves silencing *TaCFL1* genes (Figure 2A). Wax mixtures were then extracted from these wheat leaves and analyzed by gas chromatography–mass spectrometry (GC-MS). As shown in Figure 2B, loads of wax mixtures in the wheat leaves increased from 11.58 μg cm^−2^ in the BSMV-*γ* control plants to 13.27 μg cm^−2^ in the *TaCFL1*-silenced plants. Major wax constituents, including VLC alcohols, VLC alkanes, VLC aldehydes, and VLC esters, showed a remarkable increase in the wheat leaves silencing *TaCFL1* genes compared with the BSMV-*γ* control (Figure 2C). These results indicate that wheat WW domain-containing protein TaCFL1 negatively regulates the accumulation of wax. The potential regulation of TaCFL1 on the cuticle permeability of wheat leaves was analyzed by measuring water loss and chlorophyll leaching of excised wheat leaves. As shown in Figure 2D,E, decreased water loss rate and chlorophyll leaching were observed on the *TaCFL1*-silenced wheat leaves compared with the BSMV-*γ* control, suggesting that wheat leaf cuticle permeability is positively regulated by the *TaCFL1* gene. These results supported that the wheat WW domain-containing protein TaCFL1 negatively regulates the accumulation of wax contributing to the cuticle barrier property.

### 2.3. Identification of Wheat TaHDG1.1 and TaHDG1.2 Based on Homology with Arabidopsis AtHDG1 Protein

**Figure 3:**
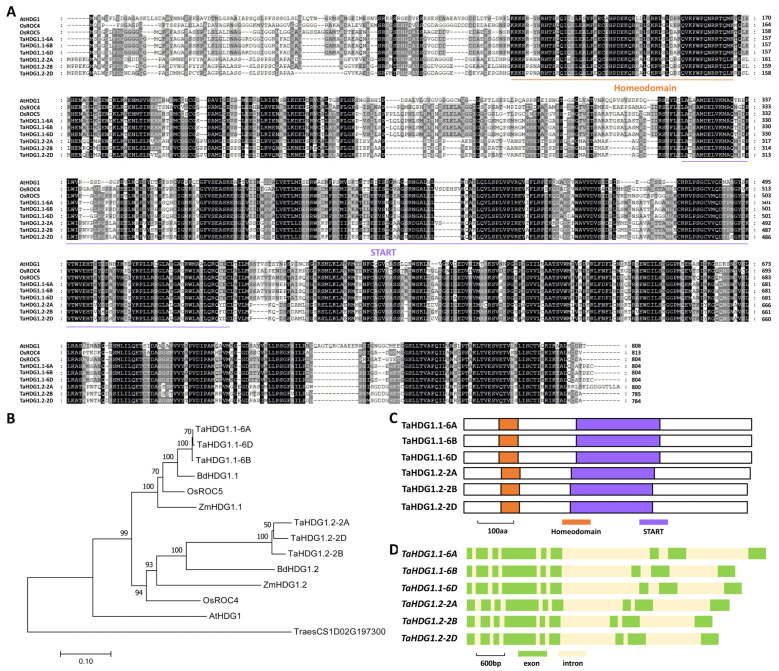
Identification of wheat TaHDG1 based on homology with Arabidopsis AtHDG1. (**A**) Protein sequence alignments of wheat TaHDG1.1, TaHDG1.2, Arabidopsis AtHDG1, rice OsROC4, and OsROC5. (**B**) Phylogenetic relationships of the HDG1 homologs from Arabidopsis, Brachypodium, maize, rice, and wheat. (**C**) Domain structures of wheat TaHDG1 proteins. (**D**) Gene architectures of wheat *TaHDG1* genes.

Arabidopsis WW domain protein AtCFL1 was demonstrated to affect the function of HD-ZIP IV transcription factor AtHDG1 in the activation of wax biosynthesis [72]. In this study, we wonder if TaHDG1, the wheat homolog of Arabidopsis AtHDG1, becomes involved in the regulation of wheat cutin and wax biosynthesis. To this end, we first employed the Arabidopsis AtHDG1 (At3g61150) as a query to search the wheat reference genome. TaHDG1.1 and TaHDG1.2 were identified as the closest wheat homologs of Arabidopsis AtHDG1. *TaHDG1.1-6A* (*TraesCS6A02G255800*), *TaHDG1.1-6B* (*TraesCS6B02G269700*), and *TaHDG1.1-6D* (*TraesCS6D02G237000*), located on wheat chromosomes 6A, 6B, and 6D, are three highly homologous sequences of the *TaHDG1.1* gene (Supplemental Figure S2). Similarly, *TaHDG1.2-2A* (*TraesCS2A02G401200*), *TaHDG1.2-2B* (*TraesCS2B02G419200*), and *TaHDG1.2-2D* (*TraesCS2D02G398600*), located on wheat chromosomes 2A, 2B, and 2D, are three highly homologous sequences of the *TaHDG1.2* gene (Supplemental Figure S3). As shown in Figure 3A, these predicted amino acid sequences of TaHDG1.1-6A, TaHDG1.1-6B, TaHDG1.1-6D, TaHDG1.2-2A, TaHDG1.2-2B, and TaHDG1.2-2D proteins shared above 47% identities with Arabidopsis AtHDG1 protein. Phylogenetic analysis further confirmed that wheat TaHDG1.1 and TaHDG1.2 are the closest wheat homologs of Arabidopsis AtHDG1 (Figure 3B). homeodomain and START domains were identified from TaHDG1.1 and TaHDG1.2 proteins (Figure 3C). Gene architecture analysis revealed that nine exons and eight introns constitute the coding regions of genomic sequences of all *TaHDG1.1* and *TaHDG1.2* genes (Figure 3D).

### 2.4. Functional Analysis of Wheat TaHDG1.1 and TaHDG1.2 in Wax Biosynthesis

**Figure 4:**
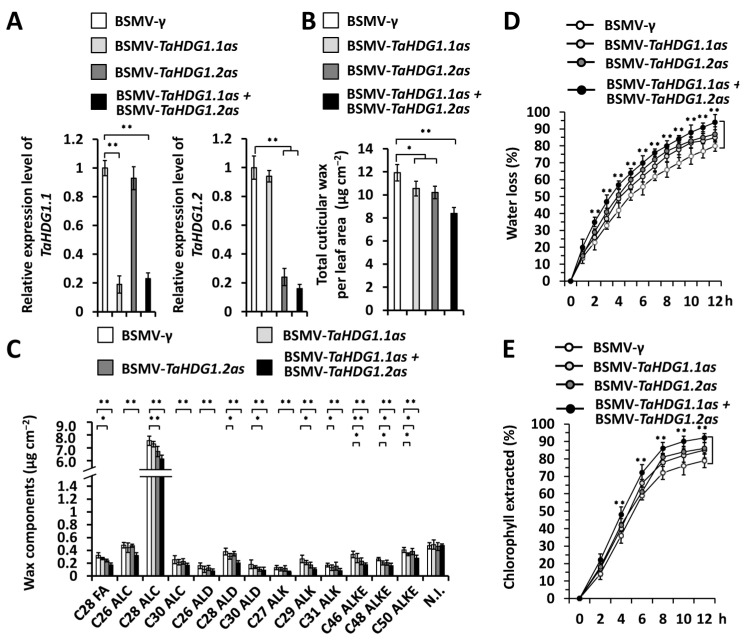
Functional analyses of wheat *TaHDG1* genes in the accumulation of leaf wax and cutin. (**A**) qRT-PCR analysis of *TaHDG1.1* and *TaHDG1.2* expression levels in the wheat leaves silencing *TaHDG1.1* (BSMV-*TaHDG1.1as*), *TaHDG1.2* (BSMV-*TaHDG1.2as*), or co-silencing *TaHDG1.1* and *TaHDG1.2* (BSMV-*TaHDG1.1as* + BSMV-*TaHDG1.2as*). (**B**) Loads of wax on the wheat leave silencing *TaHDG1.1*, *TaHDG1.2*, or co-silencing *TaHDG1.1* and *TaHDG1.2*. (**C**) Amounts of major wax components in the wheat leaves silencing *TaHDG1.1*, *TaHDG1.2*, or co-silencing *TaHDG1.1* and *TaHDG1.2*. (**D**) Water loss rates and (**E**) chlorophyll extraction levels were analyzed in wheat leaves silencing *TaHDG1.1*, *TaHDG1.2*, or co-silencing *TaHDG1.1* and *TaHDG1.2.* For (**A**–**E**), leaves of wheat plants infected with BSMV-*γ* were employed as the negative control, and three independent biological replicates per treatment were statistically analyzed, and data are presented as the mean ± SE (Student’s *t*-test; * *p* < 0.05, ** *p* < 0.01).

To explore the potential regulation of wheat *TaHDG1.1* and *TaHDG1.2* genes on the accumulation of wax and cutin, we silenced all endogenous *TaHDG1.1* or *TaHDG1.2* genes by performing BSMV-VIGS assays in the plants of the wheat cultivar Yannong 999. qRT-PCR assays demonstrated that expression levels of *TaHDG1.1* or *TaHDG1.2* genes decreased significantly in wheat leaves silencing *TaHDG1.1*, *TaHDG1.2*, or co-silencing *TaHDG1.1* and *TaHDG1.2* (Figure 4A). Wax mixtures were then extracted from these wheat leaves and analyzed by the GC-MS. As shown in Figure 4B, loads of wax mixtures in the wheat leaves decreased from 11.91 μg cm^−2^ in the BSMV-*γ* control plants to below 10.55 μg cm^−2^ in the *TaHDG1.1* or *TaHDG1.2*-silenced plants (Figure 4B). Co-silencing *TaHDG1.1* and *TaHDG1.2* genes resulted in a further reduction in wax accumulation to 8.22 μg cm^−2^ (Figure 4B). Major wax constituents, including VLC alcohols, VLC alkanes, VLC aldehydes, and VLC esters, showed a remarkable reduction in the wheat leaves co-silencing *TaHDG1.1* and *TaHDG1.2* genes compared with the BSMV-*γ* control (Figure 4C). These results indicate that wheat HD-ZIP IV transcription factors TaHDG1.1 and TaHDG1.2 partially redundantly stimulate the biosynthesis of wax. The potential regulation of TaHDG1.1 and TaHDG1.2 on the cuticle permeability of wheat leaves was analyzed by measuring water loss and chlorophyll leaching of excised wheat leaves. As shown in Figure 4D,E, enhanced water loss rate and chlorophyll leaching were observed on the *TaHDG1.1* and *TaHDG1.2*-silenced wheat leaves compared with the BSMV-*γ* control, suggesting that wheat leaf cuticle permeability is negatively regulated by *TaHDG1.1* and *TaHDG1.2* genes. These results supported that wheat HD-ZIP IV transcription factors TaHDG1.1 and TaHDG1.2 partially redundantly activate biosynthesis of wax essential for the cuticle barrier property.

### 2.5. Identification of Wheat TaKCS10 Based on Homology with Arabidopsis AtKCS10 Protein

**Figure 5:**
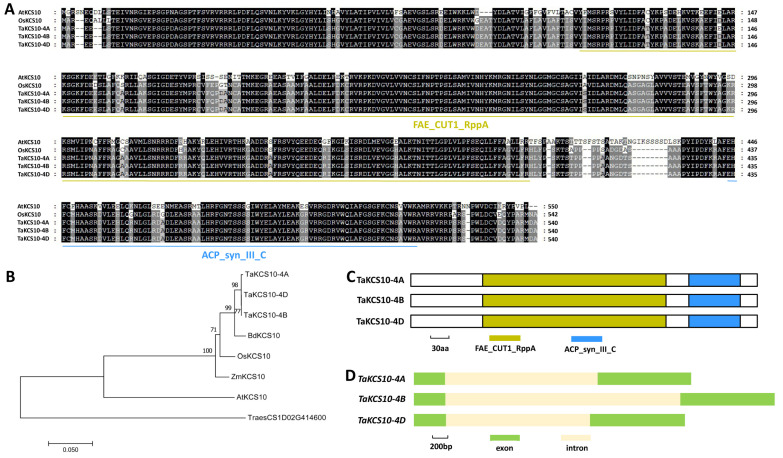
Identification of wheat TaKCS10 based on homology with Arabidopsis AtKCS10. (**A**) Protein sequence alignments of wheat TaKCS10, Arabidopsis AtKCS10, and rice OsKCS10. (**B**) Phylogenetic relationships of the KCS10 homologs from Arabidopsis, Brachypodium, maize, rice, and wheat. (**C**) Domain structures of wheat TaCFL1 proteins. (**D**) Gene architectures of wheat *TaKCS10* genes.

Arabidopsis WW domain-containing protein AtCFL1 was demonstrated to negatively regulate the expression of the 3-Ketoacyl-CoA synthase gene AtKCS10 in the suppression of wax biosynthesis [72]. In this study, we wonder if TaKCS10, the wheat homolog of Arabidopsis AtKCS10, becomes involved in wheat wax biosynthesis. To this end, we first employed the Arabidopsis AtKCS10 (At2g26250) as a query to search for the wheat reference genome. TaKCS10 was identified as the closest wheat homolog of Arabidopsis AtKCS10. TaKCS10-4A (TraesCS4A02G007400), TaKCS10-4B (TraesCS4B02G297500), and TaKCS10-4D (TraesCS4D02G296400), located on wheat chromosomes 4A, 4B, and 4D, are three highly homologous sequences of the TaKCS10 gene (Supplemental Figure S4). As shown in Figure 3A, these predicted amino acid sequences of TaKCS10-4A, TaKCS10-4B, and TaKCS10-4D proteins shared above 70% identities with Arabidopsis AtKCS10 protein. Phylogenetic analysis further confirmed that wheat TaKCS10 is the closest wheat homologs of Arabidopsis AtKCS10 (Figure 5B). FAE1/Type III polyketide synthase-like protein (FAE1_CUT1_RppA) domain and 3-Oxoacyl-[acylcarrier-protein (ACP)] synthase III C terminal (ACP_syn_III_C) domain were identified from TaKCS10 proteins (Figure 5C). Gene architecture analysis revealed that two exons and one intron constitute the coding regions of genomic sequences of all TaKCS10 genes (Figure 5D).

### 2.6. Functional Analysis of Wheat TaKCS10 in the Wax Biosynthesis

**Figure 6:**
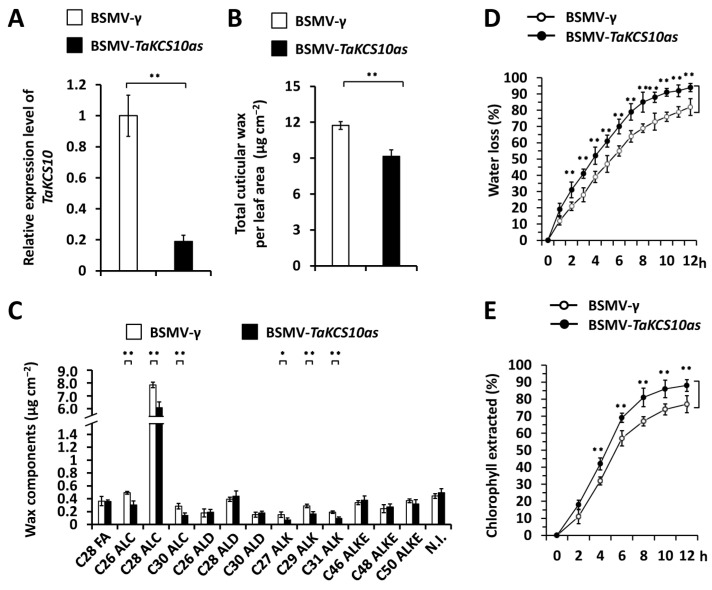
Functional analyses of wheat *TaKCS10* genes in the accumulation of leaf wax. (**A**) qRT-PCR analysis of *TaKCS10* expression levels in the wheat leaves silencing *TaKCS10* (BSMV-*TaKCS10as*). (**B**) Loads of wax on the wheat leave silencing *TaKCS10*. (**C**) Amounts of major wax components in the wheat leaves silencing *TaKCS10*. (**D**) Water loss rates and (**E**) chlorophyll extraction levels were analyzed in wheat leaves silencing *TaKCS10.* For (**A**–**E**), leaves of wheat plants infected with BSMV-*γ* were employed as the negative control, and three independent biological replicates per treatment were statistically analyzed, and data are presented as the mean ± SE (Student’s *t*-test; * *p* < 0.05, ** *p* < 0.01).

To explore the potential function of the wheat *TaKCS10* gene on the accumulation of wax, we silenced all endogenous *TaKCS10* genes by performing the BSMV-VIGS assay in the plants of the wheat cultivar Yannong 999. qRT-PCR assay demonstrated that the expression level of the *TaKCS10* gene decreased significantly in wheat leaves silencing *TaKCS10* (Figure 6A). Wax mixtures were then extracted from these wheat leaves and analyzed by the GC-MS. As shown in Figure 4B, loads of wax mixtures in the wheat leaves decreased from 11.73 μg cm^−2^ in the BSMV-*γ* control plants to below 9.16 μg cm^−2^ in the *TaKCS10*-silenced plants (Figure 6B). VLC alcohols and VLC alkanes, including C26, C28, and C30 alcohols and C27, C29, and C31 alkanes, showed a remarkable reduction in the wheat leaves silencing *TaKCS10* genes compared with the BSMV-*γ* control (Figure 6C). These results indicate that 3-Ketoacyl-CoA synthase TaKCS10 plays a key role in the biosynthesis of wax alcohols and alkanes. The potential regulation of TaKCS10 on the cuticle permeability of wheat leaves was analyzed by measuring water loss and chlorophyll leaching of excised wheat leaves. As shown in Figure 6D,E, enhanced water loss rate and chlorophyll leaching were observed on the *TaKCS10*-silenced wheat leaves compared with the BSMV-*γ* control, suggesting that wheat leaf cuticle permeability is negatively regulated by *TaKCS10* genes. These results supported that wheat 3-Ketoacyl-CoA synthase TaKCS10 functions as an essential component of wheat wax biosynthetic machinery and positively contributes to the biosynthesis of wax essential for the cuticle barrier property.

### 2.7. Transcriptional Regulation of TaKCS10 Gene by TaCFL1 and TaHDG1

**Figure 7:**
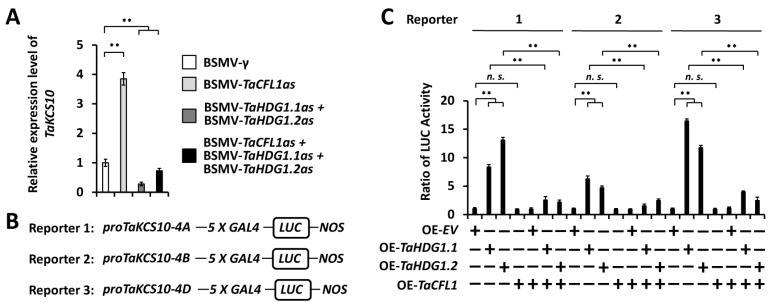
Analysis of the transcriptional regulation of *TaKCS10* genes by wheat transcriptional modulators TaCFL1, TaHDG1.1, and TaHDG1.2. (**A**) Expression levels of *TaKCS10* in the wheat leaves silencing *TaCFL1*, *TaHDG1.1*, *TaHDG1.2*, or simultaneously silencing *TaCFL1*, *TaHDG1.1*, and *TaHDG1.2* were measured by qRT-PCR assay. (**B**) Schematic depiction of the Luciferase (LUC) reporter containing promoter fragments of the *TaKCS10* gene. (**C**) Transcriptional activation of *TaKCS10* promoters by wheat transcriptional modulators TaCFL1, TaHDG1.1, and TaHDG1.2 in *Arabidopsis* protoplast cells. For A and C, three independent biological replicates per treatment were statistically analyzed, and data are presented as the mean ± SE (Student’s *t*-test; ** *p* < 0.01, *^n. s.^ p* > 0.05, *n. s.* represents no significant difference).

To examine the potential regulation of wheat WW domain protein TaCFL1 and HD-ZIP IV transcription factors TaHDG1 on the transcription of *TaKCS10,* we analyzed the expression levels of *TaKCS10* genes in the wheat leaves silencing *TaCFL1* or *TaHDG1* genes. As shown in Figure 7A, the qRT-PCR assay demonstrated that the expression level of the *TaKCS10* gene was significantly enhanced in wheat leaves silencing *TaCFL1* but remarkably reduced in wheat leaves silencing *TaHDG1*. This result indicates that TaCFL1 negatively regulates the expression of the *TaKCS10* gene, whereas TaHDG1 positively regulates *TaKCS10* expression. Notably, co-silencing of *TaCFL1* and *TaHDG1* genes resulted in the attenuated *TaKCS10* expression, resembling the silencing of *TaHDG1* genes, suggesting that upregulation of the *TaKCS10* gene in the absence of TaCFL1 relies on TaHDG1 (Figure 7A).

Thereafter, we performed the Dual-Luciferase reporter assay to examine the potential transactivation of *TaKCS10* promoters by transcription factors TaHDG1.1 and TaHDG1.2. LUC reporters harboring promoter regions of *TaKCS10-4A*, *TaKCS10-4B*, and *TaKCS10-4D* genes were co-expressed with effector proteins TaHDG1.1, TaHDG1.2, or TaCFL1 (Figure 7B). As shown in Figure 7C, overaccumulation of effector proteins TaHDG1.1 or TaHDG1.2 resulted in the LucA ratio increasing to above 4.6 from 1 for the empty vector (EV) control, suggesting that transcription factors TaHDG1.1 and TaHDG1.2 could directly activate promoters of *TaKCS10* genes. In contrast, the LucA ratio obtained from *TaKCS10* promoters was not significantly affected by over-accumulated TaCFL1 proteins (7C). Notably, the transactivation of *TaKCS10* promoters by TaHDG1.1 and TaHDG1.2 was significantly attenuated by co-expressed effector TaCFL1, suggesting that TaCFL1 attenuates the TaHDG1-mediated transcriptional activation of *TaKCS10* genes. These results collectively suggested that wheat WW domain protein TaCFL1 negatively regulates *TaKCS10* expression, probably via attenuating the TaHDG1-mediated transcriptional activation of *TaKCS10* genes.

## 3. Discussion

### 3.1. Wheat WW Domain-Containing Protein TaCFL1 Is a Negative Regulator of Wax Biosynthesis

In this study, three highly homologous *TaCFL1* genes (*TaCFL1-6A*, *TaCFL1-6B*, and *TaCFL1-6D*) separately located on the wheat chromosomes 6A, 6B, and 6D were isolated as wheat orthologs of the Arabidopsis *AtCFL1* gene. The knockdown of *TaCFL1* expression using virus-induced gene silencing results in increased accumulation of cutin and wax and decreased leaf cuticle permeability, suggesting that *TaCFL1* negatively regulates wax biosynthesis in bread wheat. Previous studies revealed that rice and Arabidopsis CFL1 negatively regulate cuticle development [72]. Toluidine blue (TB) staining demonstrated that both AtCFL1-overexpressing Arabidopsis plants and rice *cfl1* mutants are defective in cuticle development [72]. GC-MS results further showed that the total wax amount in the stems of *35S:AtCFL1* plants was significantly reduced to less than one-third of the amount accumulated on the surface of wild-type stems [72]. The amount of many wax components, such as C29 and C31 alkanes, C24, C26, C28, and C30 alcohols, and C30 fatty acid, was significantly reduced in the stems of AtCFL1-overexpressing Arabidopsis plants [72]. Consistent with these results obtained from the dicot model plant Arabidopsis, loads of total wax mixtures and major wax constituents, including VLC fatty acids, alcohols, and alkanes, showed a remarkable increase in the wheat leaves silencing the *TaCFL1* gene compared with the BSMV-γ control, suggesting that the suppression of CFL1 on wax accumulation might be conserved between dicot and monocot plants.

### 3.2. Wheat 3-Ketoacyl-CoA Synthase TaKCS10 Functions as an Essential Component of Wheat Wax Biosynthetic Machinery

Three highly homologous *TaKCS10* genes (*TaKCS10-4A*, *TaKCS10-4B*, and *TaKCS10-4D*) separately located on the wheat chromosomes 4A, 4B, and 4D were isolated as wheat orthologs of the Arabidopsis *AtKCS10* gene in this study. The silencing of *TaKCS10* expression results in a decrease in the accumulation of wax and enhances the permeability of the leaf cuticle in bread wheat. Notably, VLC alcohols and VLC alkanes, including C26, C28, and C30 alcohols and C27, C29, and C31 alkanes, showed a remarkable reduction in the wheat leaves silencing *TaKCS10* genes, suggesting that 3-Ketoacyl-CoA synthase TaKCS10 plays a key role in the biosynthesis of wax alcohols and alkanes. Arabidopsis mutant plants bearing mutations in the AtKCS10 gene show significant changes in cuticular permeability, and silencing of *MsKCS10* decreased primary alcohols and n-alkanes content in alfalfa, suggesting that the contribution of 3-Ketoacyl-CoA synthase KCS10 to wax biosynthesis might be conserved among dicot and monocot plants [73,74,75]. TaKCS1 and TaKCS6 were previously identified as 3-Ketoacyl-CoA synthases essential for wheat wax biosynthesis [76,77]. However, unlike *TaKCS10*, which specifically functions in the biosynthesis of VLC alcohols and VLC alkanes, the silencing of *TaKCS1* or *TaKCS6* resulted in reduced accumulation of all major wax constituents [76,77], indicating the functional differentiation of wheat 3-Ketoacyl-CoA synthases in the biosynthesis of wax constituents.

### 3.3. Wheat Class IV Homeodomain Transcription Factors TaHDG1.1 and TaHDG1.2 Are Partially Redundant Activators of Wax Biosynthesis

Herein, three highly homologous *TaHDG1.1* genes (*TaHDG1.1-6A*, *TaHDG1.1-6B*, and *TaHDG1.1-6D*) separately located on the wheat chromosomes 6A, 6B, and 6D, as well as three highly homologous *TaHDG1.2* genes (*TaHDG1.2-2A*, *TaHDG1.2-2B*, and *TaHDG1.2-2D*) individually located on the wheat chromosomes 2A, 2B, and 2D, were identified as wheat orthologs of the Arabidopsis *AtHDG1* gene. The Dual-Luciferase reporter assay showed that transcription factors TaHDG1.1 and TaHDG1.2 could mediate transactivation of *TaKCS10* promoters, suggesting that TaHDG1.1 and TaHDG1.2 function as transcriptional activators of the wax biosynthesis gene *TaKCS10*. A previous study showed that Arabidopsis transcription factor AtHDG1 could directly bind to the promoter region of *AtKCS10*, and the expression of *AtKCS10* was downregulated in AtHDG1 chimeric repressor plants, implicating that the direct regulation of *KCS10* expression by the HDG1 transcription factor might be conserved between the dicot plant Arabidopsis and the monocot crop bread wheat [72]. The silencing of *TaHDG1.1* or *TaHDG1.2* expression results in a decrease in the accumulation of wax and enhances the permeability of the leaf cuticle, while the co-silencing of *TaHDG1.1* and *TaHDG1.2* leads to a further decrease in the accumulation of wax, as well as a further increase in the leaf cuticle permeability, suggesting that TaHDG1.1 and TaHDG1.2 function as partially redundant activators of wax biosynthesis. Arabidopsis *hdg1* mutant showed no abnormal phenotypes in the epidermis, which might result from the functional redundancy within the Arabidopsis HD-ZIP IV gene family [72]. OsROC4 and OsROC5 are orthologs of Arabidopsis AtHDG1 in rice [78]. GC-MS analysis showed that there were more waxes in *OsROC4* overexpression rice plants, but fewer in the *roc4* mutant compared with the wild type [78]. These studies collectively suggested that HDG1 transcription factors have acquired additional functions in the wax biosynthesis during the divergence of dicots and monocots. Similarly, wheat transcription factors TaSHN1, TaKPAB1, TaEPBM1, TaMYB30, TaMIXTA1/2, and TaMYB60 were previously isolated as positive regulators of wheat wax biosynthesis, and their regulation on wax biosynthesis is divergent to some extent from their orthologs in the dicot model Arabidopsis [11,76,77,79,80,81,82].

In addition, the Dual-Luciferase reporter assay showed that TaCFL1 attenuates the TaHDG1-mediated transcriptional activation of *TaKCS10* genes, suggesting that the antagonistic interaction between CFL1 and HDG1 in the regulation of wax biosynthesis might be conserved among the dicot model plant *A. thaliana* and the monocot cereal crop bread wheat. Therefore, it is intriguing to examine the direct interaction between WW domain-containing protein TaCFL1 and HD-ZIP IV transcription factors TaHDG1.1 and TaHDG1.2 in future research. In addition, two bHLH transcription factors, CFLAP1 and CFLAP2, were demonstrated to be involved in the AtCFL1-mediated regulation of cuticle development through antagonizing HDG1 in Arabidopsis [83]. Identifying wheat orthologs of CFLAP1 and CFLAP2 and characterizing their functions in the regulation of wax biosynthesis might expand our understanding of the suppression of TaCFL1 on wheat wax biosynthesis in future research. In this study, wheat leaves silenced *TaCFL1* gene exhibited reduced water loss rate, whereas enhanced water loss rate was observed in wheat leaves silenced *TaHDG1.1*, *TaHDG1.2*, or *TaKCS10* genes. Manipulating *TaCFL1*, *TaHDG1.1*, *TaHDG1.2*, or *TaKCS10* genes using newly developed genome editing techniques could optimize wheat wax traits and provide a new avenue for stress resistance improvement in bread wheat.

## 4. Materials and Methods

### 4.1. Plant Materials and Growth Conditions

Wheat cultivar Yannong 999 and *A. thaliana* ecotype Col-0 were used as plant materials in this study. For the wheat growth, wheat seedlings planted in 300 mL pots containing nutritive medium and sterile soil (1:1, *v*/*v*) were grown in climate chambers under a 16 h light/8 h dark, 20 °C/18 °C day/night cycle, and 60% relative humidity. For the Arabidopsis growth, seeds planted in 200 mL plastic pots containing a commercial soil mix were grown in climate chambers under 16 h light/8 h dark, 23 °C, and 70% relative humidity. Wheat and Arabidopsis plants were irrigated with sterilized water, and full water-soluble compound fertilizer Huawuque was applied to maintain nutrition.

### 4.2. Gene Transient Silencing

Barley stripe mosaic virus-induced gene silencing (BSMV-VIGS) assay was performed to silence *TaCFL1, TaHDG1.1*, *TaHDG1.2*, or *TaKCS10* genes in wheat plants. Briefly, about 200 bp antisense (*as*) fragments from either the 3′ untranslated regions or coding regions of *TaCFL1, TaHDG1.1*, *TaHDG1.2*, or *TaKCS10* were amplified using primers listed in Supplemental Table S1, and PCR products were cloned into the pCa-γbLIC vector using a ligation-independent cloning technology to generate BSMV constructs BSMV-*TaCFL1as*, BSMV-*TaHDG1.1as*, BSMV-*TaHDG1.2as*, or BSMV-*TaKCS10as*. The BSMV-VIGS assay was conducted as previously described [84]. Briefly, *Agrobacterium tumefaciens* was transformed with construct DNA of pCaBS-α, pCaBS-β, and pCa-γbLIC derivatives. After suspension in the infiltration buffer (10 mM MgCl_2_, 100 µM acetosyringone, and 10 mM MES), the pCaBS-α-, pCaBS-β-, and pCa-γbLIC-carrying cell suspensions were mixed at a 1:1:1 ratio and infiltrated into the *Nicotiana benthamiana* leaves. The *N. benthamiana* sap from leaves with BSMV infection symptoms was used to inoculate the first two emerging leaves of wheat plants. *For simultaneous silencing of two genes, N. benthamiana* sap collected from leaves infected with BSMV was mixed at the 1:1 ratio for inoculation. The upper leaves with virus symptoms were harvested for the analysis of gene expression, wax composition, or leaf permeability.

### 4.3. Gene Expression Analysis

For the qRT-PCR assay, total RNA was extracted from the newly grown wheat leaves with BSMV virus symptoms about 2 weeks after BSMV infection using the EasyPure Plant RNA kit (Transgenbiotech, Beijing, China). Moreover, 1 μg RNA was treated with the TransScript one-step gDNA removal and cDNA synthesis supermix (Transgenbiotech, Beijing, China) to remove the potential DNA contamination and synthesize the cDNA template according to the manufacturer’s instructions. The Real-Time PCR assay was conducted using the CFX96 Real-Time PCR Detection System (Bio-Rad Laboratories, Hercules, CA, USA) with the qPCR Master Mix (Invitrogen, Carlsbad, CA, USA). The expression of the *TaEF1* gene was set as the reference, and the expression levels of *TaCFL1*, *TaHDG1.1*, *TaHDG1.2*, or *TaKCS10* were measured by qPCR using the qPCR Master Mix (Invitrogen) under the following programs: 95 °C for 2 min, 40 cycles at 95 °C for 20 s, 56 °C for 20 s, and 72 °C for 15 s, followed by 72 °C for 1 min. Calculation of the relative expression levels was achieved using the Bio-Rad CFX Manager 3.1 software (Bio-Rad Laboratories) with the 2^−∆∆Ct^ method, and the data were normalized to the CT values with the reference gene *TaEF1* [85] (Supplemental Table S1).

### 4.4. Wax Accumulation Analysis

Gas chromatography–mass spectrometry (GC-MS) was performed to analyze the wax accumulation in wheat leaves silencing *TaCFL1, TaHDG1.1*, *TaHDG1.2*, or *TaKCS10* genes. The GC-MS was conducted as previously described [86]. Briefly, cuticular wax mixtures were first extracted with chloroform (Merck), derivatized by reaction with bis-N,O-trimethylsilyl trifluoroacetamide, and then characterized by capillary GC (5890 Series II, Agilent Technologies, Santa Clara, CA, USA) and a flame ionization detector (6890 N, Agilent Technologies, Santa Clara, CA, USA), with a mass spectrometer (MSD 5973, Agilent Technologies, Santa Clara, CA, USA). Wax components were quantified based on flame ionization detector (FID) (6890 N, Agilent Technologies, Santa Clara, CA, USA) peak areas relative to the internal standard n-Tetracosane (Merck, Rahway, NJ, USA) per leaf area.

### 4.5. Wheat Leaf Cuticle Permeability Analysis

Water loss rates and chlorophyll extraction levels were measured to analyze the cuticle permeability of wheat leaves silencing *TaCFL1, TaHDG1.1*, *TaHDG1.2*, or *TaKCS10* genes. Water loss rates and chlorophyll extraction levels were analyzed as previously described [87]. Briefly, BSMV-VIGS wheat plants were dipped in ultrapure water for 1 h under the dark to keep the stomatal closure, and the leaves were detached. For the water loss rate analysis, weights of detached leaves were measured per hour for 12 h. For the chlorophyll leaching assay, chlorophyll was extracted from detached leaves with 80% ethanol and measured with a spectrophotometer per hour for 12 h.

### 4.6. Transcriptional Activation Analysis

A Dual-Luciferase reporter assay was performed using the *Arabidopsis* protoplast cells to analyze the transactivation of *TaKCS10* promoters by TaHDG1.1, TaHDG1.2, and TaCFL1. Promoter regions of *TaKCS10-4A*, *TaKCS10-4B*, and *TaKCS10-4D* were amplified using the primers listed in Supplemental Table S1 and cloned into the vectors 5XGAL4-LUC, and the coding regions of TaCFL1-6A, TaHDG1.1-6A, and TaHDG1.2-2A were amplified using the primers listed in Supplemental Table S1 and cloned into the vectors pRT-DBD. The Dual-Luciferase reporter assay was conducted as per the manual of the Promega Dual-Luciferase reporter assay system.

### 4.7. Statistical Analysis

For the analysis of transcriptional activation, gene expression, wax accumulation, or leaf cuticle permeability, at least three independent experiments were performed with more than three biological replicates each. Data were analyzed using Student’s *t*-test, and values represent the mean ± standard deviation (n. s *p* > 0.05, * 0.01 < *p* < 0.05, ** *p* < 0.01).

## 5. Conclusions

In this study, wheat WW domain-containing protein TaCFL1 is characterized as a negative regulator of wax biosynthesis. Silencing of *TaCFL1* results in increased wax accumulation and decreased leaf cuticle permeability. Furthermore, wheat class IV homeodomain transcription factors TaHDG1.1 and TaHDG1.2 are identified as partially redundant activators of wax biosynthesis. The silencing of *TaHDG1.1* or *TaHDG1.2* expression results in reduced accumulation of wax and enhanced leaf cuticle permeability, while the co-silencing of *TaHDG1.1* and *TaHDG1.2* leads to a further decrease in the accumulation of wax and a further increase in the leaf cuticle permeability. Moreover, wheat 3-Ketoacyl-CoA synthase TaKCS10 is isolated as an essential component of wax biosynthetic machinery. The silencing of *TaKCS10* expression results in decreased accumulation of wax and increased leaf cuticle permeability. In addition, we demonstrated that *TaKCS10* expression is activated by TaHDG1.1 and TaHDG1.2, and the TaHDG1-mediated transcriptional activation of *TaKCS10* could be attenuated by TaCFL1. Therefore, this study revealed that wheat WW domain-containing protein TaCFL1 negatively regulates wax biosynthesis, probably via attenuating the transcriptional activation of the *TaKCS10* gene mediated by partially redundant HD-ZIP IV transcription factors TaHDG1.1 and TaHDG1.2. As the interface between aerial plant organs and their environments, the waxy cuticle shields plant tissues from stressful environments. Indeed, silencing *TaCFL1* genes resulted in an attenuated water loss rate in wheat leaves. Therefore, this study could expand our knowledge of wheat wax biosynthesis and contribute to wheat breeding against drought stress.

## Data Availability

The data presented here are available upon request from correspondence.

## Supplementary Materials

The following supporting information can be downloaded at: https://www.mdpi.com/article/10.3390/ijms252313187/s1.
