# Supplementary material for: Wheat WW Domain-Containing Protein TaCFL1 Negatively Regulates Cuticular Wax Biosynthesis
Source: Int J Mol Sci. 2024 Dec 8;25(23):13187. doi: 10.3390/ijms252313187 (PMC11642047; doi:10.3390/ijms252313187)
Supplement: Supplementary file 1 [file ijms-25-13187-s001.zip › ijms-3348246-supplementary.pdf]

Supplementary Files

Wheat WW domain-containing protein TaCFL1 negatively regulates cuticular wax biosynthesis

Wanzhen Chen#, Lang Liu#, Xiaoyu Wang, Haoyu Li, Jiao Liu, Pengfei Zhi, and Cheng Chang*

College of Life Sciences, Qingdao University, Qingdao 266071, China;

# These authors contributed equally to this work

* Correspondence: cc@qdu.edu.cn

Table of Contents:

Supplemental Figure S1. Alignment of nucleotide sequences at the coding regions of allelic *TaCFL1-6A*, *TaCFL1-6B*, and *TaCFL1-6D*.

Supplemental Figure S2. Nucleotide sequences alignment at the coding regions of allelic *TaHDG1.1-6A*, *TaHDG1.1-6B*, and *TaHDG1.1-6D*.

Supplemental Figure S3. Nucleotide sequences alignment at the coding regions of allelic *TaHDG1.2-2A*, *TaHDG1.2-2B*, and *TaHDG1.2-2D*.

Supplemental Figure S4. Alignment of nucleotide sequences at the coding regions of allelic *TaKCS10-4A*, *TaKCS10-4B*, and *TaKCS10-4D*.

Supplemental Table S1. Primers used in this study.

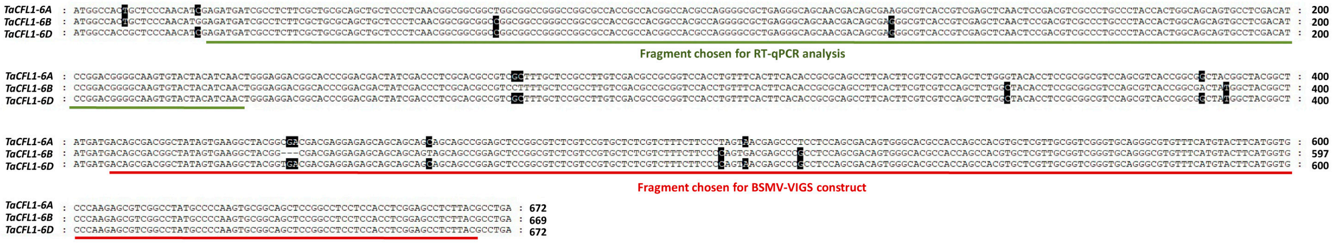

Supplemental Figure S1. Alignment of nucleotide sequences at the coding regions of allelic *TaCFL1-6A*, *TaCFL1-6B*, and *TaCFL1-6D.* The nucleotide sequences of *TaCFL1-6A*, *TaCFL1-6B*, and *TaCFL1-6D* are derived from wheat A, B and D genome, respectively. Variations among *TaCFL1-6A*, *TaCFL1-6B*, and *TaCFL1-6D* nucleotide sequences are shaded in black. Regions chosen for the *TaCFL1* silencing and expression level analysis are underlined.

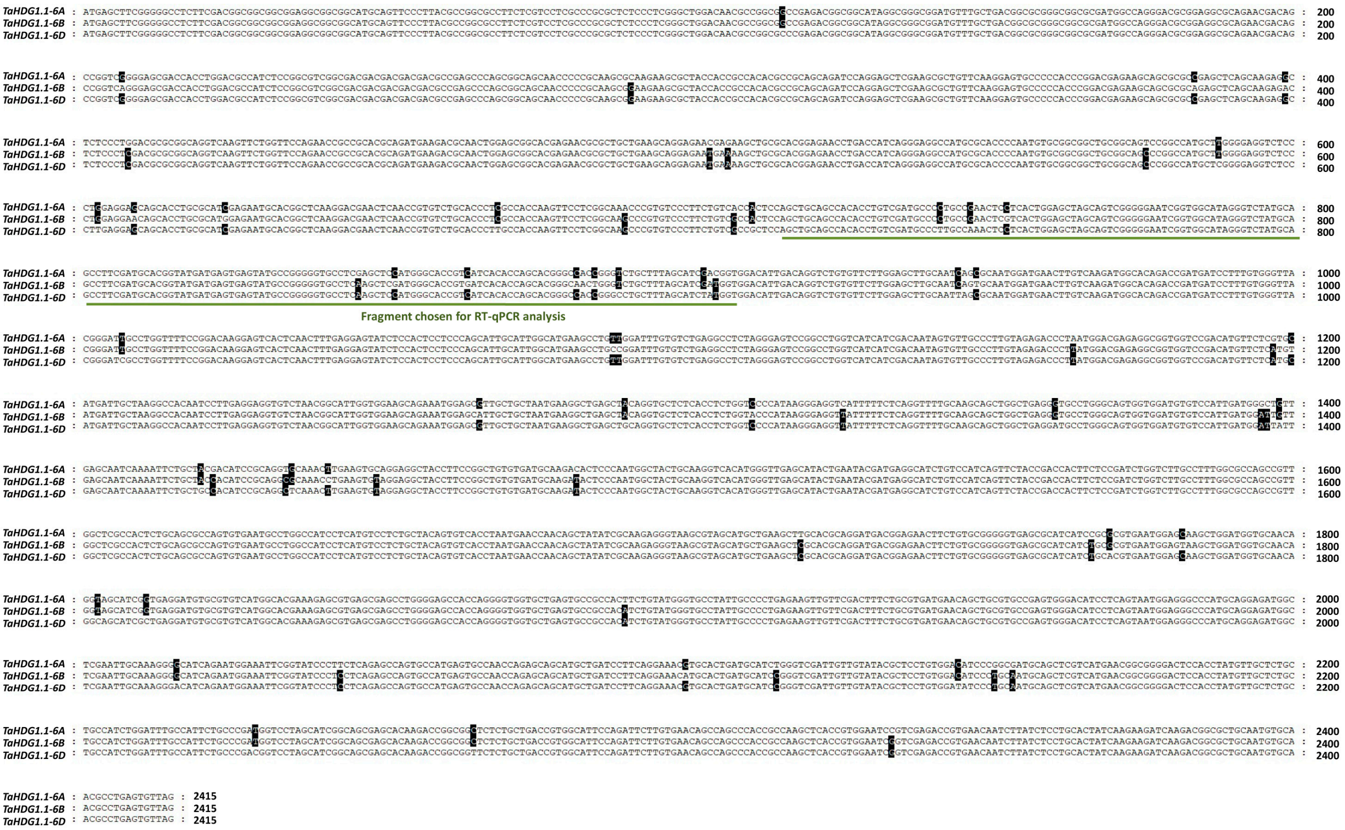

Supplemental Figure S2. Nucleotide sequences alignment at the coding regions of allelic *TaHDG1.1-6A*, *TaHDG1.1-6B*, and *TaHDG1.1-6D.* The nucleotide sequences of *TaHDG1.1-6A*, *TaHDG1.1-6B*, and *TaHDG1.1-6D* are derived from wheat A, B and D genome, respectively. Variations among *TaHDG1.1-6A*, *TaHDG1.1-6B*, and *TaHDG1.1-6D* nucleotide sequences are shaded in black. Regions chosen for the *TaHDG1.1* expression level analysis are underlined.

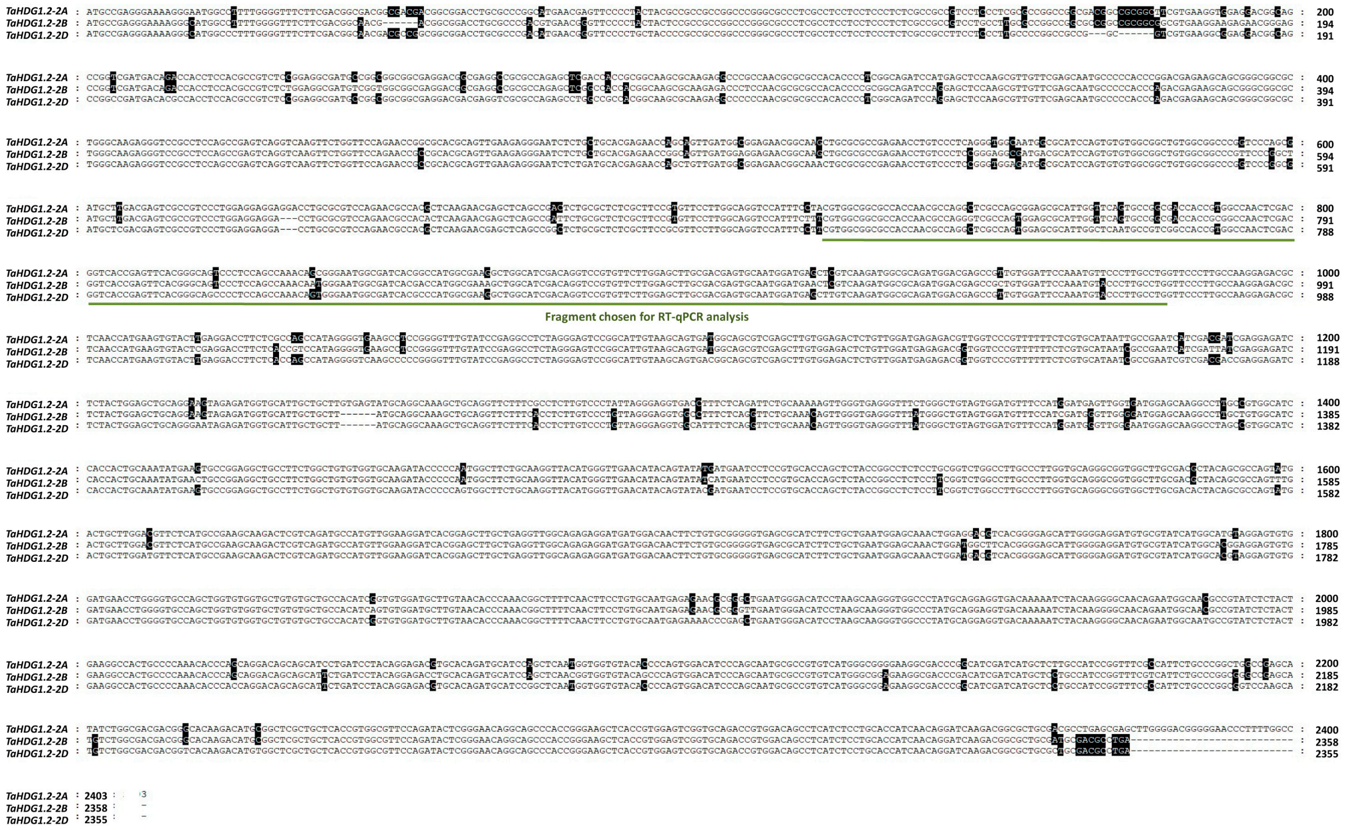

Supplemental Figure S3. Nucleotide sequences alignment at the coding regions of allelic *TaHDG1.2-2A*, *TaHDG1.2-2B*, and *TaHDG1.2-2D*. The nucleotide sequences of *TaHDG1.2-2A*, *TaHDG1.2-2B*, and *TaHDG1.2-2D* are derived from wheat A, B and D genome, respectively. Variations among *TaHDG1.2-2A*, *TaHDG1.2-2B*, and *TaHDG1.2-2D* nucleotide sequences are shaded in black. Regions chosen for the *TaHDG1.2* expression level analysis are underlined.

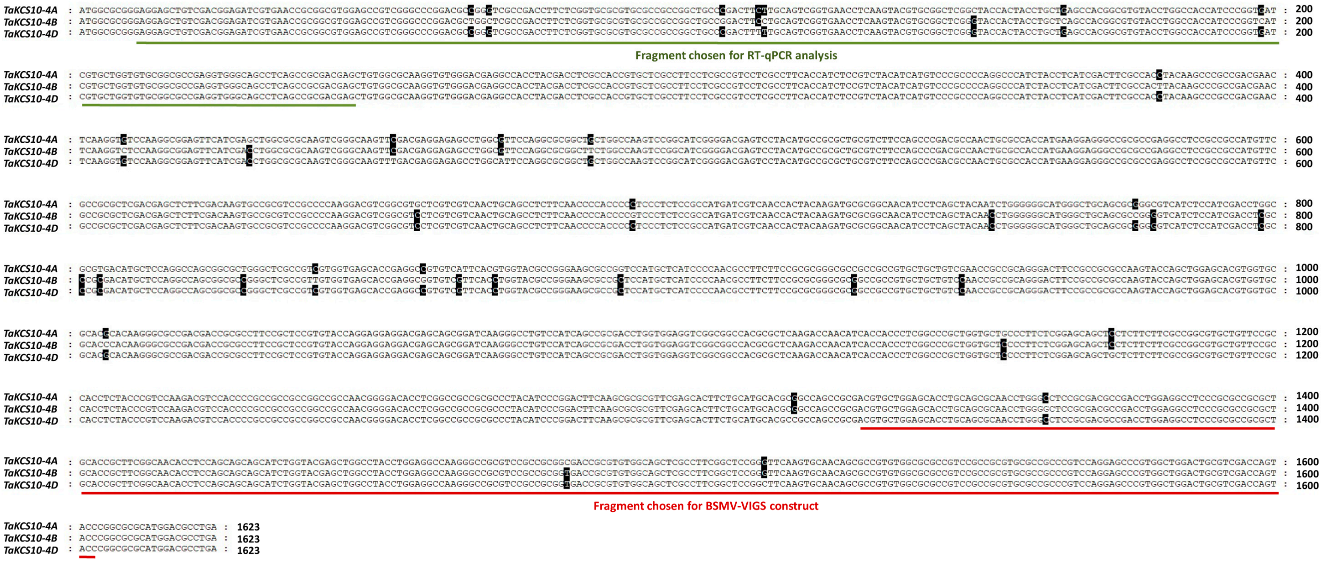

Supplemental Figure S4. Alignment of nucleotide sequences at the coding regions of allelic *TaKCS10-4A*, *TaKCS10-4B*, and *TaKCS10-4D*. The nucleotide sequences of *TaKCS10-4A*, *TaKCS10-4B*, and *TaKCS10-4D* are derived from wheat A, B and D genome, respectively. Variations among *TaKCS10-4A*, *TaKCS10-4B*, and *TaKCS10-4D* nucleotide sequences are shaded in black. Regions chosen for the *TaKCS10* silencing and expression level analysis are underlined.

Supplemental Table S1. Primers used in this study

**Primer Name**	**Sequence**	**Annotation**
qRT-PCR-*TaCFL1*-F	5' GAGATGATCGCCTCTTCG3'	qRT-PCR primer for *TaCFL1*, F primer
qRT-PCR-*TaCFL1*-R	5' GTTGATGTAGTACACTTGC3'	qRT-PCR primer for *TaCFL1*, R primer. Length of amplicons for *TaCFL1* gene used in qRT-PCR should be 207 bp.
qRT-PCR-*TaHDG1.1*-F	5'AGCTGCAGCCACACCTGTC3'	qRT-PCR primer for *TaHDG1.1*, F primer
qRT-PCR-*TaHDG1.1*-R	5'ACCGTCGATGCTAAAGCAG3'	qRT-PCR primer for *TaHDG1.1*, R primer. Length of amplicons for *TaHDG1.1* gene used in qRT-PCR should be 194 bp.
qRT-PCR-*TaHDG1.2*-F	5' CGTGGCGGCGCCACCAAC3'	qRT-PCR primer for *TaHDG1.2*, F primer
qRT-PCR-*TaHDG1.2*-R	5' CAGGCAAGGGAACATTTG3'	qRT-PCR primer for *TaHDG1.2*, R primer. Length of amplicons for *TaHDG1.2* gene used in qRT-PCR should be 257 bp.
qRT-PCR-*TaKCS10*-F	5' GAGGAGCTGTCGACGGAG3'	qRT-PCR primer for *TaKCS10*, F primer
qRT-PCR-*TaKCS10*-R	5' CTCGTCGCGGCTGAGGCTG3'	qRT-PCR primer for *TaKCS10*, R primer. Length of amplicons for *TaKCS10* gene used in qRT-PCR should be 237 bp.
qRT-PCR-*TaEF1*-F	5' CAGGACGTTTACAAGATTG3'	qRT-PCR primer for *TaEF1*, F primer
qRT-PCR-*TaEF1*-R	5' CAAAACCACGCTTCAGATC3'	qRT-PCR primer for *TaEF1*, R primer. Length of amplicons for *TaEF1* gene used in qRT-PCR should be 223 bp.
pCa-*TaCFL1as*-F	5'AAGGAAGTTTAACGTAAGAG GCTCCGAGGTG3'	For construct of BSMV-*TaCFL1as*, F primer
pCa-*TaCFL1as*-R	5'AACCACCACCACCGTGACAG CGACGGCTATAGTG3'	For construct of BSMV-*TaCFL1as*, R primer
pCa-*TaHDG1.1as*-F	5'AAGGAAGTTTAACAAAAATA AATCCCAGTTG3'	For construct of BSMV-*TaHDG1.1as*, F primer
pCa-*TaHDG1.1as*-R	5'AACCACCACCACCGTACATTT CTTGAAGGACAT3'	For construct of BSMV-*TaHDG1.1as*, R primer
pCa-*TaHDG1.2as*-F	5'AAGGAAGTTTAACTTGCTGGA TACCATTCT3'	For construct of BSMV-*TaHDG1.2as*, F primer
pCa-*TaHDG1.2as*-R	5'AACCACCACCACCGTGACAG ACAGTTGGAGGCTA'	For construct of BSMV-*TaHDG1.2as*, R primer
pCa-*TaKCS10as*-F	5'AAGGAAGTTTAAGTACTGGTC GACGCAGTC3'	For construct of BSMV-*TaKCS10as*, F primer
pCa-*TaKCS10as*-R	5'AACCACCACCACCGTGACGTG CTGGAGCACCTGC'	For construct of BSMV-*TaKCS10as*, R primer
pENTRY-*proTaKCS10-4A*-F	5'GGGGACAAGTTTGTACAAAA AAGCAGGCTTCGTTGGTAGCAG TAGTTTAG3'	For the construction of pENTRY-*proTaKCS10-4A*, F primer
pENTRY-*proTaKCS10-4A*-R	5'GGGGACCACTTTGTACAAGA AAGCTGGGTCGCTCGACTGGTG GCGGCGA3'	For the construction of pENTRY-*proTaKCS10-4A*, R primer. Length of amplicons for *TaKCS10-4A* promoter fused to *LUC* should be 736 bp.
pENTRY-*proTaKCS10-4B*-F	5'GGGGACAAGTTTGTACAAAA AAGCAGGCTTCGTTGCTGACCA AGTTCAG3'	For the construction of pENTRY-*proTaKCS10-4B*, F primer
pENTRY-*proTaKCS10-4B*-R	5'GGGGACCACTTTGTACAAGA	For the construction of pENTRY-*proTaKCS10-4B*, R primer.

	AAGCTGGGTCGACTGGTGGTGGGCAGTG3'	Length of amplicons for *TaKCS10-4B* promoter fused to *LUC* should be 956 bp.
pENTRY-*proTaKCS10-4D*-F	5'GGGGACAAGTTTGTACAAAAAAGCAGGCTTCGGAGAGCATTTTGGTGGC3'	For the construction of pENTRY-*proTaKCS10-4D*, F primer
pENTRY-*proTaKCS10-4D*-R	5'GGGGACCACTTTGTACAAGAAAGCTGGGTCGACTGGTGGTGGGCGGTG3'	For the construction of pENTRY-*proTaKCS10-4D*, R primer. Length of amplicons for *TaKCS10-4D* promoter fused to *LUC* should be 1008 bp.
pENTRY-*TaCFL1*-F	5'GGGGACAAGTTTGTACAAAAAAGCAGGCTTCATGGCCACTGCTCCCAACAT3'	For the construction of pENTRY-*TaCFL1*, F primer
pENTRY-*TaCFL1*-R	5'GGGGACCACTTTGTACAAGAAAGCTGGGTCTCAGGCGTAAGAGGCTCC3'	For the construction of pENTRY-*TaCFL1*, R primer
pENTRY-*TaHDG1.1*-F	5'GGGGACAAGTTTGTACAAAAAAGCAGGCTTCATGAGCTTCGGGGGCCTCT3'	For the construction of pENTRY-*TaHDG1.1*, F primer
pENTRY-*TaHDG1.1*-R	5'GGGGACCACTTTGTACAAGAAAGCTGGGTCCTAACACTCAGGCGTTGCA3'	For the construction of pENTRY-*TaHDG1.1*, R primer
pENTRY-*TaHDG1.2*-F	5'GGGGACAAGTTTGTACAAAAAAGCAGGCTTCATGCCGAGGGAAAAGGGA3'	For the construction of pENTRY-*TaHDG1.2*, F primer
pENTRY-*TaHDG1.2*-R	5'GGGGACCACTTTGTACAAGAAAGCTGGGTCCTAGGCCAAAAGGGTTCC3'	For the construction of pENTRY-*TaHDG1.2*, R primer
